# Comparison of Clinical and Biomechanical Outcomes between Partial Fibulectomy and Drug Conservative Treatment for Medial Knee Osteoarthritis

**DOI:** 10.1155/2019/4575424

**Published:** 2019-10-30

**Authors:** Guo Chen, Bin Xu, Jinwei Xie, Yong Nie, Shuo Tang, Jun Ma, Qiang Huang, Zongke Zhou, Bin Shen, Xia Li, Hai Shen, Fuxing Pei

**Affiliations:** ^1^Sichuan Provincial Orthopedic Hospital, Chengdu 610041, Sichuan, China; ^2^Department of Orthopedic Surgery, West China Hospital, Sichuan University, Chengdu 610041, Sichuan Province, China; ^3^Department of Orthopedics, The Eighth Affiliated Hospital, Sun Yat-sen University, Shenzhen 518035, Guangdong Province, China

## Abstract

**Background:**

Upper partial fibulectomy has been preliminarily proved to have the efficacy for pain alleviation and improvement of function in patients with mild to moderate medial compartment knee osteoarthritis (KOA). However, the previous studies lack the control group with other treatments. The aim of this prospective, randomized controlled study is to compare the clinical and biomechanical effects between upper partial fibulectomy and drug conservative treatment on improvement of clinical pain, function, and gait for patients with mild to moderate medial knee osteoarthritis (KOA) and further discuss its biomechanical mechanism.

**Methods:**

From August 2016 to February 2017, 49 and 48 patients with mild to moderate medial KOA were allocated to fibulectomy and drug groups. We assessed the patients' visual analog scale (VAS) pain score, Hospital for Special Surgery (HSS) knee score, limb alignment, passive flexion/extension range of motion (ROM) of the knee, and 3D gait kinematics and kinetics parameters before and after intervention. Repeated-measures ANOVA with Dunnett's post hoc assessment and multivariate analysis of variance were applied for intragroup and intergroup comparisons, respectively.

**Results:**

The improvement in the fibulectomy group on the VAS pain score, HSS knee score, walking speed, and walking knee range of motion (ROM) was statistically better than that in the drug group. The decreased overall peak knee adduction moment (KAM) (decreased by 16.1%) and hip-knee-ankle (HKA) angle (decreased by 0.99° from a more varus alignment to a more neutral alignment) of the affected and operated side 1 year after surgery were observed in the fibulectomy group.

**Conclusion:**

This research demonstrated that as a biomechanical intervention, upper partial fibulectomy can be a better choice in pain relief and function and gait improvement than drug conservative treatment for patients with early-stage knee OA. The long-term clinical outcomes, indication, and rationale for the improvement in clinical symptoms should be investigated further.

## 1. Introduction

About 13% of women and 10% of men aged 60 years and older suffer from symptomatic knee osteoarthritis (KOA) [1], which lowers quality of life and presents a considerable burden to the health services as it causes pain and loss of function. Age, genetic susceptibility, obesity, female gender, trauma, muscle weakness, joint laxity, mechanical forces, and meniscal injuries are risk factors for KOA [1]. The load of medial and lateral compartments of the knee is unbalanced. The pressure bearing by the medial compartment was 2.2 times larger than that by the lateral compartment during walking [2]. Greater medial load was theorized to fasten the progression of articular cartilage degradation and increase the risk of medial KOA disease [3]. Patients with mild to moderate KOA exhibited significant pain alleviation and function improvement by several kinds of conservative treatments including valgus knee brace [4], lateral wedge insoles [5], and gait modification [6]. However, mixed reviews existed as their effectiveness has not been demonstrated by high-quality, randomized controlled clinical trials. Except for conservative treatment, some invasive procedures were also used for treatment. High tibial osteotomy (HTO) is an established surgical procedure in selected patients with medial KOA. However, HTO is reported to be associated with a moderate frequency of complications which included nonunion or delayed union, under- or overcorrection of the deformity, peroneal nerve palsy, implant failure, recurrence of varus deformity, and loss of correction [7]. General complications associated with this procedure include infection (2.3–54.5%), deep vein thrombosis (l.3–9.8%), vascular injury (0.4%), and compartment syndrome [7]. Total knee arthroplasty (TKA) and unicompartmental knee arthroplasty (UKA) are also alternatives for moderate- to end-stage KOA [8], but complications such as postoperative infection, periprosthetic fracture, aseptic loosening caused by periprosthetical osteolysis, and subsequent revision especially for younger KOA patients spurred us to figure out other options.

A recently proposed new surgery, upper partial fibulectomy, has been preliminarily proved to have the efficacy for pain alleviation and improvement of the medial joint space and function in patients with medial KOA [9, 10]. Besides, our previous pilot studies further demonstrated the effectiveness of upper partial fibulectomy for patients with moderate radiographic medial KOA using clinical assessment, 3D gait analysis, finite element analysis, and dynamic lower limb musculoskeletal analysis [10, 11]. However, part of the previous researches were retrospective, had no control group, and lacked sufficient quantitative evidence, which had only low-level evidence.

Given that the patients receiving HTO suffered more severity of KOA than patients who received drug conservative treatment or upper partial fibulectomy, we conducted this prospective, randomized controlled study to compare the clinical and biomechanical effects between drug conservative treatment and upper partial fibulectomy on improvement of clinical pain and function for patients with mild to moderate medial compartment KOA and further discuss its potential biomechanical mechanism.

## 2. Methods

### 2.1. Participants

This prospective, randomized controlled study was approved by the Institutional Review Board of West China Medical Center of Sichuan University (No. 2016-200) before patient enrolment, and the study was registered in the International Clinical Trial Registry. Written informed consent and research authorization were obtained from all patients. From August 2016 to February 2017, community-dwelling individuals with mild to moderate medial compartment KOA were screened for inclusion in the study. According to the standard of American College of Rheumatology classification criteria for knee osteoarthritis [12], an experienced orthopedic surgeon made the diagnosis of KOA. The following are the criteria for the inclusion of patients: (1) distinctive radiological features congruous with medial compartment-dominant KOA according to the Kellgren–Lawrence grade (grades I–III) [13] and (2) patients reporting of medial aspect-dominant pain of the knee. Exclusion criteria included the following: (1) walking ability limited more by comorbidity than KOA (cardiopulmonary disease, arteriosclerosis obliterans, etc.); (2) medical history of knee operation or intra-articular injection or acupuncture within 6 months; (3) systemic use of corticosteroids within 6 months; (4) surgical history of the index knee including total knee arthroplasty, high tibial osteotomy, and fracture internal fixation; and (5) KOA with a valgus deformity.

The sample size calculation was based on the anticipated difference in pain between two groups at 1-year follow-up. According to our pilot study, we anticipated the VAS pain score of the fibulectomy group would decrease to around 1 point at 1-year follow-up. Assuming that the VAS pain score of the drug group would drop to around 2 points based on research on nonsurgical management of knee osteoarthritis [14], a power of 95%, and a significance level of 5%, the required sample size was 26 patients in each group. Considering the fact that following the increasing number of patients who are recruited in the trial is the decrease of both type I and type II errors, we recruited all the patients who met the inclusion criteria from August 2016 to February 2017.

### 2.2. Study Design and Treatment Management

Recruited patients were randomized to either the fibulectomy group or the drug group. Randomization was blind and performed with the use of sealed envelopes in a 1 : 1 ratio opened just prior to treatment. After signing the informed consent, patients were invited to a gait laboratory. Assessment including pain grade, physical function, lower extremity alignment, and 3D gait analysis was conducted preoperatively. One investigator (GC) measured the clinical indices, and a single biomechanical expert (YN) conducted 3D gait analysis. They were both blinded to the subgroup and intervention. One month, 3 months, 6 months, and one year after receiving intervention, participants were invited to the laboratory for follow-up including the same assessment conducted preoperatively.

**Figure 1:**
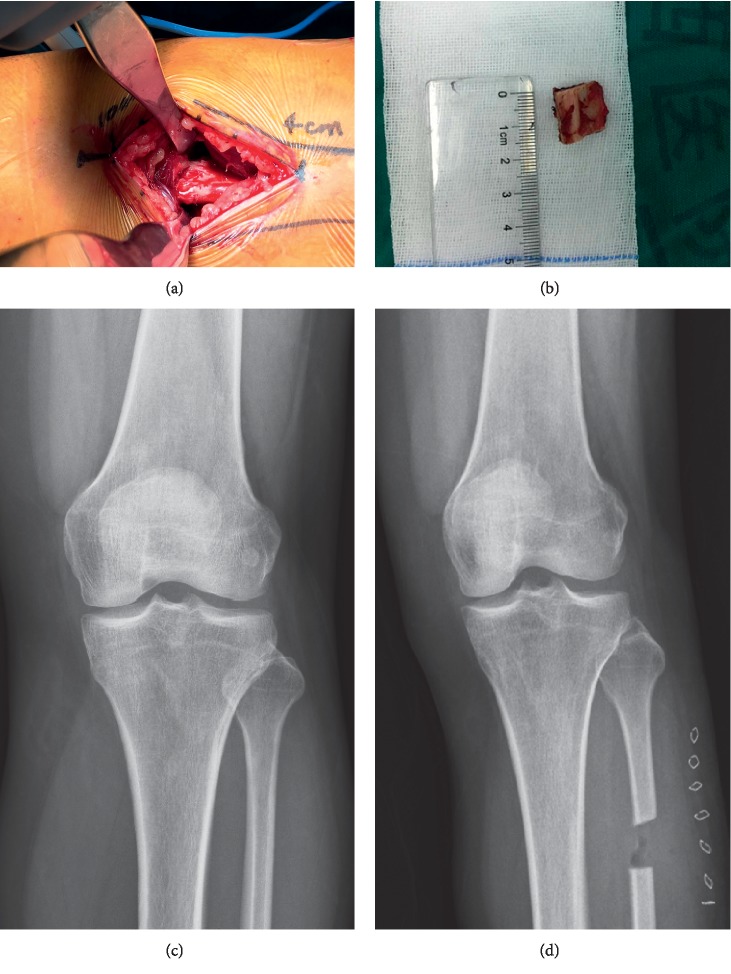
(a) Intraoperative clinical image showing an incision carried out along the fibula between 4 and 10 cm from the fibular head. The upper section of the fibula was exposed from the intermuscular space by tissue separation. (b) An osteotomy of 1 cm was performed between 4 and 6 cm from the proximal fibular head. (c, d) Knee radiograph before and after upper partial fibulectomy.

Patients in the drug group were admitted to community hospitals and received drug conservative treatment under the instruction and supervision of the community doctor. Oral diclofenac sodium (50 mg, twice a day) was administrated for 1 to 2 months. When the pain was relieved and patients felt that they did not need to have their intake, the drug could be withdrawn. Patients in the fibulectomy group received upper partial fibulectomy with the standard procedure [10]. A senior surgeon orthopedist (FXP) performed all the operation in the same operation room, composed of four senior orthopedic surgeons. After local anesthesia using 2% lidocaine, an incision was made between 4 and 10 cm from the fibular head along the fibula. The posterolateral crural intermuscular septum (between the soleus and peroneus longus/peroneus brevis) was identified after soft tissue was released. After exposing the upper part of the fibula from the intermuscular space, gauze was put between the soft tissue and the fibula to separate them, thus avoiding potential risks of neurovascular injuries. The fibulectomy of 1 cm was conducted between 4 and 6 cm from the proximal fibular head (Figures 1(a) and 1(b)). Bone wax was used to enclose the broken end of the fibula. After rinse, hemostasis, and incision closure, an elastic bandage was wrapped around the incision to achieve local compression. The anteroposterior radiographs before and after operation are shown in Figures 1(c) and 1(d). Patients were discharged from the hospital on the second day after surgery. They could walk freely without a walking aid and return to normal life when no signs of significant pain or local swelling are found. They also received oral diclofenac sodium (50 mg bid) for 15–30 days. The standard of drug withdrawal was the same as that in the drug group.

All the patients were guided as follows: (1) The straight-leg testing was carried out (100–200 times per day, 5 s per time) to improve quadriceps strength. (2) Reducing food intake and regular aerobic exercises (swimming, biking, etc.) were encouraged to lose weight when BMI ≥30 kg/m^2^. (3) The walking distance should be limited to less than 5 kilometers when pain occurred during walking and less than 10 kilometers when pain was relieved. (4) Climbing and going upstairs or downstairs should be avoided during daily life.

### 2.3. Pain Assessment, Physical Function Assessment, Limb Alignment Measurement, and 3D Gait Analysis

The specific methods and instruments by which we conducted pain (visual analog scale (VAS) pain score) and physical function (Hospital for Special Surgery knee score and passive flexion/extension range of motion) assessment, limb alignment (hip-knee-ankle (HKA) angle) measurement (measuring on long-limb radiograph), and 3D gait analysis could be seen in our previous research [10, 11].

### 2.4. Statistical Analysis

One investigator (GC) performed the data statistics who was blinded to the subgroup and intervention. All data analysis was performed by SPSS version 22 (SPSS Inc., USA). A repeated-measures ANOVA with Dunnett's post hoc assessment was applied for the intragroup comparison of clinical and 3D gait outcomes. Multivariate analysis of variance (MANOVA) was applied for the intergroup comparison of clinical and 3D gait outcomes. Student's *t*-test, chi-square test, or Wilcoxon's rank-sum test was used to analyze demographic data between the two groups. The statistical difference was defined as *P* < 0.05.

## 3. Results

**Figure 2:**
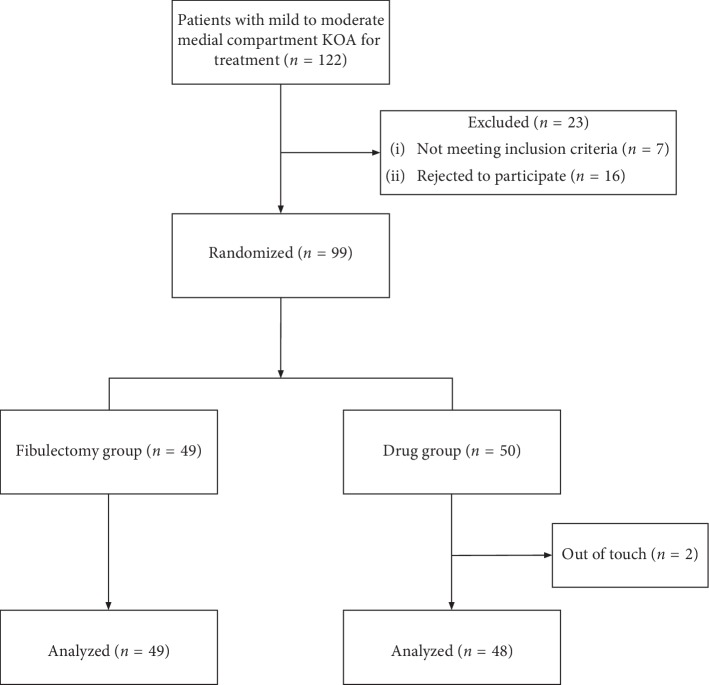
Flow diagram of the patients involved.

During the recruitment period from August 2016 to February 2017, 122 patients with mild to moderate medial compartment KOA were admitted to our hospital for treatment. Of them, seven patients were ineligible and sixteen patients declined participation. The remaining 99 eligible participants were recruited and formed the study cohort. Forty-nine were randomized to the fibulectomy group and fifty to the drug group. Two cases in the drug group were lost to follow-up. Therefore, 49 cases in the fibulectomy group and 48 cases in the drug group were included in the final study and analysis (Figure 2).

No statistical difference was found with regard to the sex, age, height, weight, BMI, duration, and Kellgren–Lawrence grade between the two groups. The operation time of upper partial fibulectomy was 26.39 ± 10.16 minutes, and the length of hospital stay was 3.80 ± 1.35 days (Table 1). No adverse events were observed, such as cardiovascular and cerebrovascular events, pulmonary embolism, and death. Similarly, there were no surgery-related complications either, such as incision infection and peroneal nerve injury.

For patients receiving upper partial fibulectomy, the average hospitalization expenses are 6588.41 ± 375.55 RMB and the average postoperative length of stay is 1.51 ± 0.51 days. After an average of 15.20 ± 3.01 days postoperatively, all these 49 patients receiving upper partial fibulectomy went back to work. Forty-eight of them went back to the previous work. Only one patient, whose was a porter, changed his work and became a security guard in a community.

### 3.1. Clinical Outcomes

**Figure 3:**
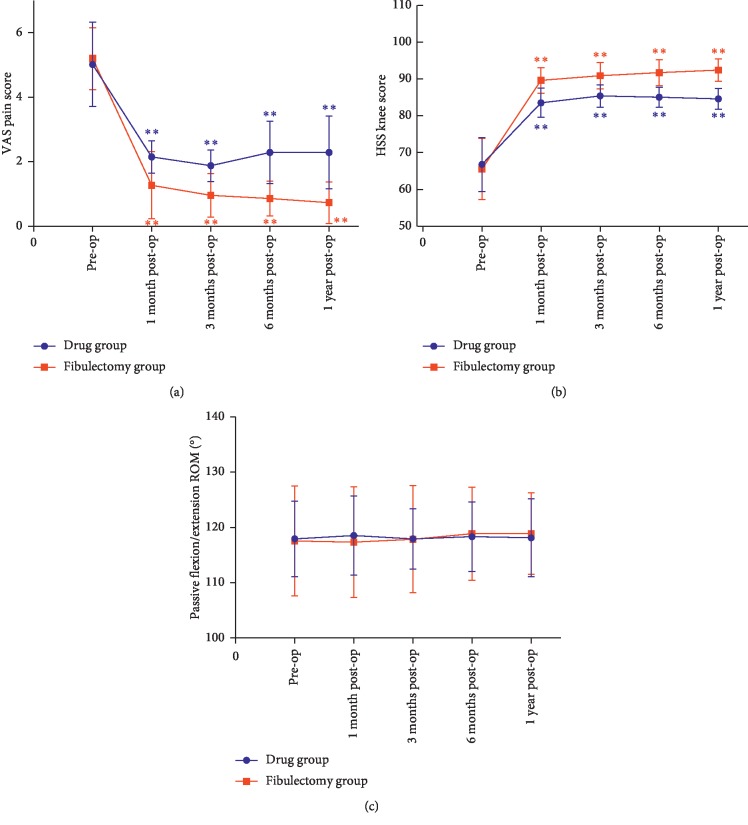
Changing trend of clinical outcomes before upper partial fibulectomy and during the 1-year follow-up. (a) VAS pain score. (b) HSS knee score. (c) Passive flexion/extension ROM. Blue ^*∗*^ denotes a significant difference compared with the preoperative value in the drug group (^*∗*^ denotes *P* < 0.05; ^*∗∗*^ denotes *P* < 0.001). Red ^*∗*^ denotes a significant difference compared with the preoperative value in the fibulectomy group (^*∗*^ denotes *P* < 0.05; ^*∗∗*^ denotes *P* < 0.001). VAS: visual analog scale; HSS: Hospital for Special Surgery; ROM: range of motion.

No statistical difference was found in the VAS pain score and HSS knee score before treatment between the two groups (*P* > 0.05). The VAS pain score was lower in the fibulectomy group than in the drug group at 1 month (*P* < 0.001) and was still lower in the fibulectomy group at 3 months, 6 months, and 1 year (*P* < 0.001). For intragroup comparison, the VAS pain score was lower in every postoperative follow-up time compared with the preoperative value for both drug and fibulectomy groups (*P* < 0.001). The HSS knee score in the fibulectomy group was higher than that in the drug group at 1 month (*P* < 0.001) and was still higher in the fibulectomy group at 3 months, 6 months, and 1 year (*P* < 0.001). For intragroup comparison, the HSS knee score was higher in every postoperative follow-up time compared with the preoperative value for both drug and fibulectomy groups (*P* < 0.001). No statistical difference was found in the passive flexion/extension knee ROM before and after treatment between the two groups and between each group (*P* > 0.05). In the following year after treatment, the incidence of TKA in the drug group was higher than that in the fibulectomy group (5/48 vs 0/49, *P* < 0.05). The clinical outcome comparison between groups and within groups is shown in Table 2 and Figure 3.

### 3.2. 3D Gait Analysis

No statistical difference was found before treatment between the two groups in walking speed, cadence, walking knee ROM, the overall peak knee adduction moment (KAM), and HKA angle. The walking speed and walking knee ROM were better in the fibulectomy group than in the drug group at one-year follow-up (*P* < 0.05). The KAM and HKA angle were better in the fibulectomy group than in the drug group since the first month and were still better than those in the drug group at one-year follow-up (*P* < 0.05). No statistical difference was found in cadence after treatment between two groups (*P* > 0.05). The results of the intergroup comparison are shown in Table 3.

**Figure 4:**
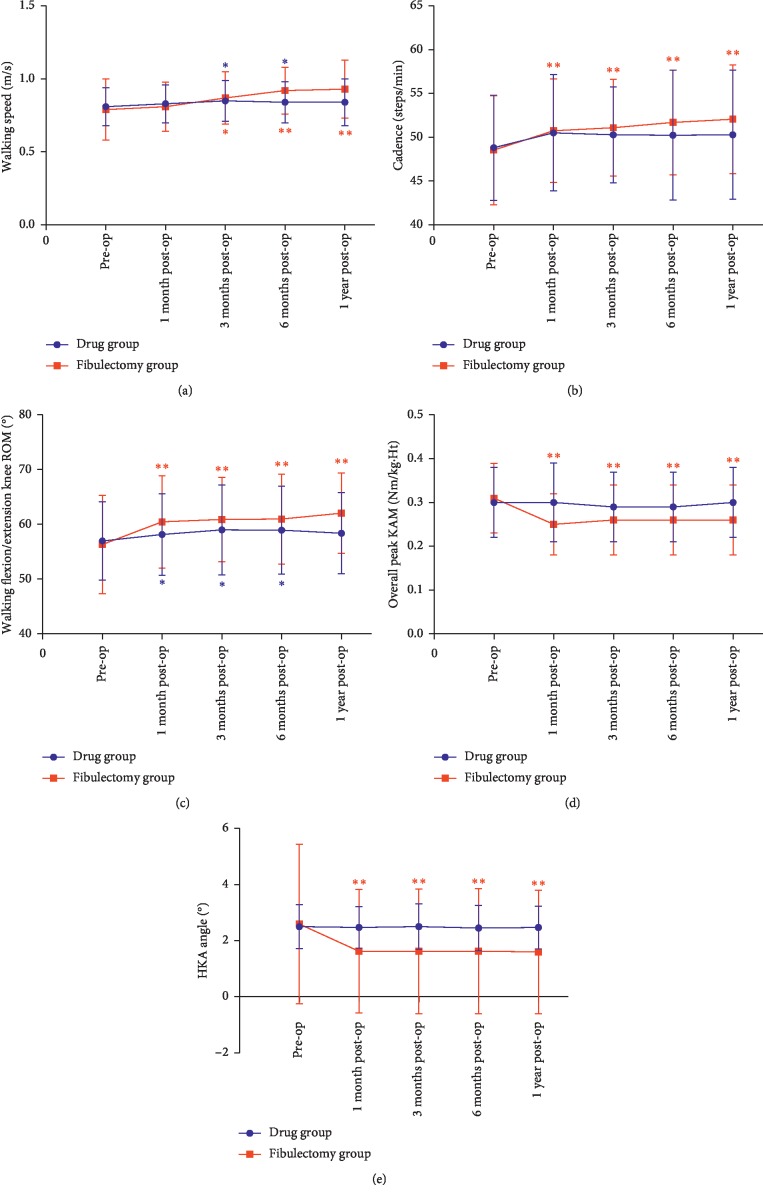
Changing trend of lower limb alignment and 3D gait outcomes before upper partial fibulectomy and during the 1-year follow-up. (a) Gait speed. (b) Cadence. (c) Walking flexion/extension ROM. (d) Overall peak KAM. (e) HKA angle. Blue ^*∗*^ denotes a significant difference compared with the preoperative value in the drug group (^*∗*^ denotes *P* < 0.05; ^*∗∗*^ denotes *P* < 0.001). Red ^*∗*^ denotes a significant difference compared with the preoperative value in the fibulectomy group (^*∗*^ denotes *P* < 0.05; ^*∗∗*^ denotes *P* < 0.001). ROM: range of motion; KAM: knee adduction moment; HKA angle: hip-knee-ankle angle.

In the fibulectomy group, compared with pretreatment, the walking speed increased at 3 months after treatment (*P* < 0.01) and was still higher at 6-month and 1-year follow-up (*P* < 0.001). For cadence, walking knee ROM, KAM, and HKA angle in the fibulectomy group, the indexes measured at every follow-up time postoperatively were all better than those before treatment (*P* < 0.05). In the drug group, the promotion of walking speed and ROM could be observed 3 months and 6 months postoperatively (*P* < 0.05). However, these benefits were not inspected at one-year follow-up (*P* > 0.05). The results of intragroup comparison of fibulectomy and drug groups are shown in Figure 4.

## 4. Discussion

The practicability and potential benefits of upper partial fibulectomy treating patients with medial KOA have been demonstrated by current studies [9–11]. This randomized controlled trial also showed that pain relief, function improvement, more efficient gait, more neutral alignment, and lower medial knee loading could be obtained within 1 year after fibulectomy compared with drug treatment. Furthermore, fibulectomy is minimally invasive with low cost and short hospital stay and scarcely produces any adverse effects [9–11]. Thus, upper partial fibulectomy represents an important treatment strategy for step therapy of KOA.

Pain is a ubiquitous symptom in KOA, leading to disability and loss of autonomy in seniors. Adequate pain relief and function improvement were closely related to postoperative satisfaction. At different follow-up time, we observed that the pain was significantly ameliorated after treatment of patients in both groups. This finding supports other published reports of upper partial fibulectomy for those with medial KOA [9–11]. Furthermore, the pain relief in the fibulectomy group is better than that in the drug group at different follow-up time with statistical differences. The VAS pain score of upper partial fibulectomy decreased by 86.0% compared with the decrease of 42.4% [5], 40.4% [5], 9.0% [4], 44.4% [6], and 43.3% [15] reported for treatments with lateral wedge insoles, acupuncture, knee braces, toe-out gait modification, and opening wedge HTO, respectively. Therefore, despite the heterogeneity in inclusion criteria, baseline values of VAS pain scores, follow-up, etc. among these study cohorts, these researches still demonstrated the effectiveness of upper partial fibulectomy on pain relief.

Constant function improvement could be observed in terms of the HSS knee score and gait parameters for patients receiving upper partial fibulectomy [10, 11]. In this research, though the HSS knee score improved in both groups after treatment, the amelioration of the HSS knee score in the fibulectomy group was better than that in the drug group at last follow-up with statistical differences. In our study, the HSS knee score in the partial fibulectomy group increased by 40.9% compared with increases of 30.7% [5], 29.4% [5], 17.3% [16], 28.5% [6], and 49.2% [17] reported for treatment with lateral wedge insoles, acupuncture, knee braces, toe-out gait modification, and opening wedge HTO, respectively. With regard to improvement of gait efficiency after upper partial fibulectomy, gait parameters including walking speed, cadence, and active flexion/extension knee ROM increased significantly, and this improvement could be noted at 1-year follow-up as well. As to other ways of treatment, Birmingham et al. [18] demonstrated a 5.5% increase of walking speed after HTO at 2-year follow-up, however with 2 patients requiring revision surgery for nonunion. Similarly, knee braces could improve walking speed and cadence of patients, but they reduced the overall sagittal-plane ROM by increasing maximal knee flexion during the stance phase and also reducing knee extension during the swing phase, with patients complaining of uncomfortable superstructure and non-user-friendly designs [19]. Hence, compared with HTO and knee braces, upper partial fibulectomy could improve function and gait with better patient compliance and no risk of nonunion.

While walking, medial tibiofemoral compartment loading can be correspondingly reflected by KAM [20]. Previous literature studies reported KAM during gait has been correlated with medial KOA pain, varus alignment, mechanical axis, load distribution, compartmental bone mineral content, and radiographic evidence of disease progression [19, 20]. Our previous research provided further support for the overall peak KAM as an effective surrogate for limb alignment [10]. Similarly, the HKA angle, measured on a full leg radiograph in the standing position, is another index representing limb alignment which was defined as the angle between the mechanical axes of the femur and the tibia. It was regarded as neutral alignment when the HKA angle is between 1 and 1.5 degrees [12]. Our findings showed a mean 16.1% reduction of the overall peak KAM for the affected (operated) side in the fibulectomy group at last follow-up. However, the KAM decrease was not found in the drug group. Furthermore, the TKA incidence in the fibulectomy group at 1-year follow-up was smaller than that in the drug group (*P* < 0.05). On the contrary, the HKA angle in the fibulectomy group improved 1 month after surgery, and the melioration of HKA angle was maintained at 1-year follow-up, while no improvement of HKA angle could be seen in the drug group. Compared with previous HTO reports, we found a relatively small degree of HKA angle improvement in patients receiving upper partial fibulectomy [21]. We attributed this to the fact that, before surgery, the patients for HTO had larger HKA angle than those for upper partial fibulectomy in our study (preoperative HKA angle in HTO vs fibulectomy patients: 5.8 ± 2.4 [22] vs 2.59 ± 2.85 and 6.0 ± 2.6 [21] vs 2.59 ± 2.85). As the patients receiving HTO had larger HKA angle improvement postoperatively, HTO presented larger KAM improvement than upper partial fibulectomy. To summarize, our findings suggested that 10–20% reduction of the overall peak KAM and a more neutral alignment (reflexed by HKA angle improvement), as achieved in this study by upper partial fibulectomy, contribute to slowing down of KOA progression and delaying the time to take TKA, thus relatively avoiding complications after TKA [8].

The mechanism by which upper partial fibulectomy improves clinical pain, function, and gait has been preliminarily investigated in our previous studies [10, 11]. We found that the load of the tibia was redistributed postoperatively. The stress of the medial region of the tibial plateau decreased (decreased by 19.7%) significantly after upper partial fibulectomy. We hypothesized that, after upper partial fibulectomy, the increased muscle activity of the biceps femoris causes a competition between the biceps femoris and the peroneus, leading to displacement of the fibular head. The displacement creates a tension in the lateral knee that may cause the observed improvement in HKA angle from a more varus alignment to a more neutral alignment. Therefore, the improvement of alignment and medial knee loading contributes to pain relief and improvement of gait and function.

Based on our observation on the clinical effect of upper partial fibulectomy and the exploration of its mechanism using 3D gait analysis, finite element knee joint model analysis, and dynamic lower limb musculoskeletal analysis [10, 11], we found that upper partial fibulectomy improved limb alignment passively and indirectly within a relatively small range by competition between muscles. However, HTO improved the mechanical axis actively and directly to a large extent through wedge osteotomy. Accordingly, elder patients with less muscle-adjusting ability may probably not obtain a desirable clinical effect after upper partial fibulectomy. Similarly, it might be difficult to correct the mechanical axis of patients who had a more serious varus alignment or a larger HKA angle preoperatively by upper partial fibulectomy. Thus, we proposed the indications of upper partial fibulectomy as follows: (1) age less than 70 years, (2) mild varus malalignment or HKA angle less than 5 degrees, (3) mild to moderate medial compartment KOA, (4) joint stability, and (5) good ROM and nonobese. These indications could be a preliminary guidance for patient selection and for ensuring success of surgery. We will further explore more precise indications to guide clinical practice.

Except for intrinsic limitations usually occurring in motion analysis researches (i.e., variability in gait measurements because of body anthropometrics and independent skin displacement, definition of the neutral position, and time of gait studies), our study presented with some limitations: First, the follow-up was relatively short. Since biomechanical and clinical benefits of fibulectomy might vary during the natural course of the disease, a longer duration may be needed to characterize and to better understand the biomechanical and clinical benefits and limitations of upper partial fibulectomy. Second, though we have control group receiving drug treatment, a sham surgical group is needed to assess fibulectomy in a truly blinded and nonbiased way. Third, we recruited patients with medial compartment-dominant KOA of grades I–III based on the Kellgren–Lawrence grade, so the results may not be applicable to patients in the late stages of OA. Fourth, a bias toward better outcomes of upper partial fibulectomy exists as a greater placebo effect was observed by some researches for more invasive procedures [23]. Further studies comparing upper partial fibulectomy with other invasive procedures such as HTO are planned to be conducted. Finally, though we tried to explore the mechanism of the promising biomechanical and clinical benefits of fibulectomy using 3D gait analysis, finite element knee joint model analysis, and dynamic lower limb musculoskeletal analysis in our previous research [10, 11], the exact mechanisms of load changes on the tibia and whole joint needed further and deeper investigation.

In summary, this research demonstrated that the effects of upper partial fibulectomy on pain relief and function and gait improvement are better than those of drug conservative treatment for patients with early-stage knee OA. The long-term clinical outcomes, indication, and rationale for the improvement in clinical symptoms should be investigated further in the future.

## Figures and Tables

**Table 1:** Baseline characteristics and intraoperative data.

	Drug group	Fibulectomy group	*P* value
Cases	48	49	
Sex^‡^			
Male	6	5	0.721
Female	42	44	
Affected^‡^			
Left	24	23	0.763
Right	24	26	
Age (years)^†^	56.06 ± 8.75	57.86 ± 9.51	0.336
Height (cm)^†^	158.38 ± 6.91	157.82 ± 6.96	0.693
Weight (kg)^†^	61.44 ± 8.65	62.10 ± 8.80	0.709
BMI (kg/m^2^)^†^	24.64 ± 4.20	25.10 ± 4.39	0.595
Course (years)^†^	3.69 ± 1.53	3.98 ± 1.45	0.349
KL grades^∆^			
1	10	6	0.159
2	32	33	
3	6	10	
Surgical time (min)	—	26.39 ± 10.16	—
LOH (days)	—	3.80 ± 1.35	—

BMI: body mass index; KL grades: Kellgren–Lawrence grades; LOH: length of hospital stay; ^‡^Pearson's chi-square test; ^†^Student's *t*-test; ^∆^Wilcoxon's rank-sum test.

**Table 2:** Intergroup comparison of clinical outcomes.

	Drug group	Fibulectomy group	*P* value
VAS pain score^§^			
Before treatment	5.02 ± 1.31	5.20 ± 0.96	0.433
After 1 month	2.15 ± 0.50	1.27 ± 1.04	<0.001
After 3 months	1.88 ± 0.49	0.96 ± 0.68	<0.001
After 6 months	2.29 ± 0.97	0.86 ± 0.54	<0.001
After 1 year	2.29 ± 1.13	0.73 ± 0.64	<0.001
HSS knee score^§^			
Before treatment	66.81 ± 7.32	65.57 ± 8.29	0.437
After 1 month	83.54 ± 3.97	89.63 ± 3.46	<0.001
After 3 months	85.38 ± 3.08	90.90 ± 3.56	<0.001
After 6 months	85.04 ± 2.75	91.71 ± 3.54	<0.001
After 1 year	84.58 ± 2.83	92.39 ± 3.03	<0.001
Passive knee ROM^§^ (°)			
Before treatment	117.92 ± 6.83	117.55 ± 9.95	0.834
After 1 month	118.54 ± 7.14	117.35 ± 10.01	0.501
After 3 months	117.92 ± 5.44	117.86 ± 9.68	0.970
After 6 months	118.33 ± 6.30	118.88 ± 8.43	0.720
After 1 year	118.13 ± 7.04	118.88 ± 7.38	0.609
TKA incidence^∂^	5/48	0/49	0.027

VAS: visual analog scale; HSS: Hospital for Special Surgery; passive knee ROM: passive flexion/extension range of motion of the knee joint; ^§^multivariate analysis of variance; ^∂^Fisher's exact test.

**Table 3:** Intergroup comparison of the 3D gait analysis results.

	Drug group	Fibulectomy group	*P* value
Speed^§^ (m/s)			
Before treatment	0.81 ± 0.13	0.79 ± 0.21	0.671
After 1 month	0.83 ± 0.13	0.81 ± 0.17	0.643
After 3 months	0.85 ± 0.14	0.87 ± 0.18	0.634
After 6 months	0.84 ± 0.14	0.92 ± 0.16	0.011
After 1 year	0.84 ± 0.16	0.93 ± 0.20	0.016
Cadence^§^ (steps/min)			
Before treatment	48.81 ± 6.00	48.53 ± 6.24	0.820
After 1 month	50.50 ± 6.65	50.75 ± 5.91	0.848
After 3 months	50.29 ± 5.49	51.09 ± 5.53	0.367
After 6 months	50.23 ± 7.42	51.70 ± 5.97	0.286
After 1 year	50.28 ± 7.38	52.06 ± 6.21	0.202
Walking knee ROM^§^ (°)			
Before treatment	56.96 ± 7.15	56.34 ± 9.01	0.707
After 1 month	58.16 ± 7.44	60.47 ± 8.43	0.156
After 3 months	58.99 ± 8.24	60.91 ± 7.70	0.238
After 6 months	58.95 ± 8.00	60.99 ± 8.21	0.217
After 1 year	58.39 ± 7.41	62.04 ± 7.35	0.017
Overall peak KAM^§^ (Nm/kg·Ht)			
Before treatment	0.30 ± 0.08	0.31 ± 0.08	0.539
After 1 month	0.30 ± 0.09	0.25 ± 0.07	0.007
After 3 months	0.29 ± 0.08	0.26 ± 0.08	0.030
After 6 months	0.29 ± 0.08	0.26 ± 0.08	0.057
After 1 year	0.30 ± 0.08	0.26 ± 0.08	0.004
HKA angle^§^ (°)			
Before treatment	2.50 ± 0.78	2.59 ± 2.85	0.831
After 1 month	2.47 ± 0.74	1.62 ± 2.20	0.013
After 3 months	2.50 ± 0.81	1.62 ± 2.22	0.011
After 6 months	2.45 ± 0.81	1.62 ± 2.23	0.017
After 1 year	2.47 ± 0.76	1.60 ± 2.20	0.011

Walking knee ROM: walking flexion/extension range of motion of the knee joint; KAM: knee adduction moment; HKA angle: hip-knee-ankle angle; ^§^multivariate analysis of variance.

## Data Availability

The original data supporting the results of this study are available from the corresponding author upon request if needed.
